# Fracture of the Nasal Bones

**Published:** 1848-07

**Authors:** 


### ARTICLE VIII.

**The following cases are reported in the *London Lancet* as having occurred at the Middlesex Hospital.**

### *Fracture of the Nasal Bones.*

The nose, from its exposed position, is not unfrequently injured by blows or falls, the cartilages being separated from the bones, or the bones themselves fractured. Although fracture of the nasal bones, being necessarily the result of direct violence, is generally attended by considerable tumefaction and disfigurement from effusion of blood, it is seldom that any serious consequences result from such an accident. Occasionally, however, it happens, that blows on this organ, which may perhaps have occasioned but slight external injury, are followed by symptoms indicative of inflammation of the brain or its investing membranes. This arises from the connection between the septum of the nose and that portion of the floor of the skull formed by the cribriform plate of the ethmoid bone. A blow on the nose may force up the septum, and fracture this delicate plate of bone. The possibility of such an occurrence is alluded to by most systematic writers on surgery, but is usually spoken of rather as an accident which might happen, than as one which is likely to be met with in practice. Within the last few months, four cases of injury of the face, with fracture of the nasal bones, have been admitted into the Middlesex Hospital. Three of these cases have terminated fatally, from inflammation of the membranes of the brain; and in all three there was found fracture of the cribriform plate of the ethmoid. In two of these fatal cases, the visible injury done to the nose was so trifling, that fracture of the ossa nasi was not suspected during life. The danger in these cases is not to be estimated by the amount of external injury. It is dependent, not on the degree of force producing the injury, but on the direction in in which that force is applied. The nose may be completely crushed by a blow acting laterally or from above, and the cribriform plate escape uninjured; but a much slighter blow, so applied as to drive the septum directly upwards, may fracture the cribriform plate from which it depends, and the result may be death from inflammation of the membranes of the brain. It is probable that accidents of this kind are much more frequent than is generally suspected, as, from the nature of the lesion, it is very likely to be overlooked in the hurried manner in which post-mortem examinations are frequently conducted.

The cases are related in the order in which they occurred.

*Fracture of the Nasal Bones, and of the Cribriform Plate of the Ethmoid; Inflammation of the Membranes of the Brain; Death.*

Case 1. J. T——, a stableman, aged thirty, of intemperate habits, was admitted late on the evening of the 15th of December, having just before been kicked in the face by a horse. He walked as steadily and answered as rationally as could be expected of a man who had evidently been drinking pretty freely. He had a vertical, irregular, lacerated wound, completely dividing the upper lip on the left side, and over the frontal sinus, a little to the left of the median line, was a cut about an inch in length; the bone beneath was denuded, and a portion of it driven in and partially detached, so as to admit the point of the little finger. No other injury, with the exception of some apparently slight bruises, was detected at this time. The patient, who seemed somewhat flushed and excited, apparently from drink, was sent to bed, the wound in the lip united by a suture, water-dressing applied to the forehead, and he was ordered a full dose of calomel and jalap. On the following day he complained much of pain in the head, which was still unrelieved on the 17th, although the bowels had been freely opened. The face was now much swollen, but without any redness, the skin being unusually pale. The eyelids were completely closed from œdema. Ordered two grains of calomel every six hours.

*Dec.* 18th.—Still complains of pain in the head, but the face is less swollen. About the middle of the day he began to talk incoherently, and attempted to get out of bed; towards night he became worse, being more muddled and confused, and his pulse, which had been quick, was now slow, but with some degree of power, and very irregular, both in time and force. He was at this time perfectly quiet, lying on his back. It was observed, that the right hand, which had been raised from the bed to admiit of the pulse being felt, retained its position after the support of the surgeon's hand had been withdrawn. The arm was now fully extended; it retained its position. The left arm was now tried; it also retained any position it was made to assume. Both arms were kept elevated and fully extended, for some minutes, in various positions, no attempt being made by the patient, whe remained perfectly passive during these experiments, to move the limbs; but after a time he drew his arms under the clothes, and curled himself up in the bed, in the position of a patient with concussion of the brain. During the time the arms were extended, there were slight convulsive twitches of the muscles. As the motions were reported to have been extremely dark and offensive, and as there was considerable power in the pulse, a strong dose of purgative medicine was ordered immediately.

19th.—He had not slept, and had been very restless, rambling a good deal, and frequently attempting to get out of bed. The bowels had acted freely, and the motions were more natural in appearance. He was in the morning much clearer in his intellect, answering questions quite rationally. The pulse was again regular, but weak. Omit calomel. To take porter and beef-tea. Towards the evening the delirium returned, and he became so noisy and troublesome, that it was necessary to remove him from the ward into a private room. At 11 P. M. he was bathed in a most profuse sweat; his face colored, but not flushed; his breathing short and hurried; his pulse excessively quick and feeble; his tongue dry, and coated with reddish-brown fur. He was incessantly talking, but scarcely made any attempts to get out of bed. As, from the present symptoms and the previous history of the patient, this was considered as a pure case of delirium tremens, he was ordered a pint of porter, with half a drachm of the liquor opii Indiensis, (Jeremie's solution of opium,) which, after a good deal of persuasion, he swallowed. No effect appears to have been produced by this dose; he continued talking during the remainder of the night, and until about 7 o'clock on the following morning, when he became much quieter, and in about half an hour after expired; four days and a half after the accident, and about forty hours after the first appearance of delirium.

21st.—The body was examined thirty hours after death. The face was considerably discolored from bruises. On reflecting back the skin covering the upper part of the face, a small portion of bone was found driven in over the frontal sinus, on the left side, corresponding to the wound in the integument. A small quantity of sero-purulent fluid was found in the cavity of the arachnoid. Beneath the arachnoid, which was thickened and opaque, was a purulent deposit covering the entire surface of the brain and cerebellum. The substance of the brain was healthy, but more than usually vascular; the veins on its surface were extremely tortuous. On removing the dura mater, the cribriform plate of the ethmoid bone was found to be fractured and driven up, and a fissure extended backwards through the sphenoid bone to the right anterior clinoid process. The ossa nasi being now examined were found to be both fractured. This had not been discovered during life. The viscera of the chest and abdomen were not examined.

### *Fracture of the Ossa Nasi, with Considerable Contusion—Recovery.*

Case 2.—W. H. B——, a cabman, aged about 30; admitted Dec. 24th. Had been laid up for several weeks with some disease of his chest. The first day he resumed his work, he was thrown from his seat, and fell on his face. There was no wound, but the face was much bruised, particularly about the nose, which was greatly swollen and discolored. On taking hold of the nose, it was found to be perfectly movable, the movements being accompanied by distinct crepitus. Although the bony supports of the nose were so extensively fractured, there was but little displacement, and nothing required to be done to maintain the parts in a proper position. The patient was kept in bed on low diet, and cold lotion was applied to the face. No unfavorable symptoms occurred; the swelling gradually subsided, and he was discharged a few weeks afterwards, retaining but slight traces of the accident. This case, which may be taken as an example of the ordinary fracture of the ossa nasi, is recorded, from its affording an instructing contrast with the previous one. In Case 1, the apparent injury done to the nose was so slight as to escape detection during life; but the force, though producing such slight external injury, has been so applied as to drive up the bony septum of the nose, and break in fragments the delicate plate of bone which separates the cavity of the nose from the membrane of the brain. In Case 2, the visible injury was considerable, but was confined to the vault of the nose, and the accident was followed by no symptoms indicative of internal injury. The septum either yielded, or the force was not applied in such a direction as to drive it up against the cribriform plate.

*Lacerated Wounds of the Face and Scalp—Extensive Fracture of the Bones of the Face—Fracture of the Cribriform Plate of the Ethmoid—Death.*

Case 3.—A powerful muscular man, aged 54, was brought to the hospital, February 25th, at 5 P. M. He had been riding on the shaft of his cart, close by the hospital, but being drunk, he had slipped off, and the wheel of the cart, from the statement of those who accompanied him, passed over his head. He did not appear to be at all stunned, or to suffer any pain or inconvenience, and walked with a firm step from the surgery into the ward. On examination, it was found that there was a semilunar flap of scalp, about four inches across, torn up on the upper part of the head, a small portion of bone being divested of its periosteum. There was a lacerated wound on each side of the median line, over the root of the nose. The wound on the right side, much the largest, extended down to the bone, exposing the margin of the orbit, and was prolonged to the free border of the upper eyelid, dividing, however, merely the skin covering the lid. The point of the finger introduced into this wound could be passed out into the orbit beneath the roof, which was divested of its periosteum. The eye was not burst, but blood appeared effused in its interior, and the sight was destroyed. The wound on the left side, at the inner extremity of the brow, was much less extensive, but extended to the bone, which was felt to be fractured into the frontal sinus. On the left cheek there was an irregular cut along the center, and another straight cut extended along the line of junction between the nose and the cheek. There was also a deep cut at the lower part of the wing of the nose on the same side. The ossa nasi and the ascending portions of the superior maxillary bones were felt to be fractured. The flap of scalp, which bled but little, was easily brought into position, and retained by strapping. The wounds on the face were united by sutures, and lint, dipped in cold water, was kept continually applied to the face. The oozing of blood from the numerous wounds was for some hours very considerable—a good deal passed backwards into the mouth; but by continuing the application of cold, it was, about 4 P. M., completely checked.

*Feb.* 26th.—Doing well; no bleeding; no complaint of pain; pulse rather thick; thirsty. Ordered an aperient, to be followed by salines, with antimony.

27th.—Passed a good night; slight symptoms of erysipelas appeared about the face; pulse much weaker. Omit salines; wine, four ounces; beef-tea. In the evening much the same; but complained of pain in the right shoulder. Had not previously complained of pain there, or, indeed, anywhere else. The shoulder was examined; but no bruise or other injury was detected.

28th.—Appeared going on very favorably during the day, and when visited late at night, was sleeping soundly, but he had been dull, and had not spoken during the evening. About two A. M. on the following morning, a change suddenly took place; he woke up, and his breathing became very laborious, noisy, and hurried, each inspiration being accompanied by a loud snorting sound. It was difficult to make out the seat of this first sound, and impediment to the entrance of air. At first, it was conjectured that the larynx was the part in fault, but on raising the patient up in bed, so as to place him nearly in the sitting posture, his breathing became much freer, and less hurried, and it was now found, that the noise during inspiration was in a great part occasioned by the air passing through the wound over the left brow, during the respiratory movements, the nose being probably closed by clotted blood. On holding a candle near the wound, which communicated with the frontal sinus, the flame was observed to be drawn towards the wound in inspiration, and during expiration the air rushed out with such force as nearly to extinguish the light. At this time, he appeared perfectly insensible, and incapable of speaking. Although he was kept supported in the same position, the difficulty of breathing again came on in the course of a few hours; he became gradually weaker, and died at half-past 7 A. M., about five hours after he was first siezed with difficulty of breathing, and three days and a half after the accident.

*Examination of the body, thirty hours after death. Head:* The nasal bones, the nasal spine of the frontal, and the ascending processes of the superior maxillary bones, were broken across and driven downwards, so as to completely detach them from the frontal bone. On the right side, the external angular process of the frontal bone was broken off; the malar bone completely separated; and the zygoma fractured. The soft parts were much contused, with considerable effusion of blood, particularly into the substance of the right temporal muscle. The flap of the scalp was not united, but there was pus between it and the bone. There was considerable vascularity of the whole surface of the membranes of the brain, with slight extravasations of blood, especially on the inferior surface of both the cerebrum and cerebellum. Yellow, fetid pus was spread over the surface of the arachnoid, covering the right hemisphere; and greenish colored lymph was found in the pia mater, beneath the arachnoid, on the left side. The brain was firm and vascular; a moderate quantity of clear serum was contained in the ventricles. On removing the dura mater, which was entire, the cribriform plate of the ethmoid was found to be completely shattered. *Neck:* A considerable extravasation of blood was found in the cellular tissue beneath the skin, on the anterior and right side of the neck, extending inwards to the root of the tongue. The larynx, trachea, and œsophagus, appeared quite healthy. *Thorax:* Heart and pericardium quite healthy; right lung much congested, and œdematous; left lung united to the walls of the chest by numerous old adhesions; the lower lobe breaking down under the fingers in the attempt to remove it; its tissues much softened, infiltrated with dark blood, and very fetid; in a state of incipient gangrene; it floated in water. This condition was confined to the lower lobe; the upper appeared healthy. On examining the right shoulder, where he complained of pain on the evening of the 27th, no external mark of injury was observed; but pus was found in considerable quantity in the bursa beneath the deltoid, extending down over the long head of the biceps, but external to the proper sheath; there was no pus in the cavity of the joint. The left shoulder and both knee-joints were examined, but they appeared quite healthy. The viscera of the abdomen were also quite healthy.

***Fracture of the Nasal Bones and of the Cribriform Plate of the Ethmoid Bone; Inflammation of the Membranes of the Brain; Death.***

Case 4. Thomas T——, aged forty-eight, came to the hospital in the afternoon of March 22nd, stating that he had just before fallen from a height of eight or ten feet. There was some ecchymosis around the left eye, and a small lacerated wound over the margin of the orbit, but no fracture could be felt. As he was said to have been stunned for some minutes after he fell, he was, as a measure of precaution, taken into the hospital, although at the time of his admission there were no symptoms indicative of any injury to the brain. At night he complained of pain in the chest, and of some difficulty in breathing, but this he stated to be a complaint he had been subject to for years. He was ordered a dose of calomel and colocynth.

On the following morning, the 23d, there did not appear to be much the matter with him, but about the middle of the day he complained of feeling cold and chilly, and the nurse gave him an additional blanket; soon after this a great change took place in his appearance, his countenance became vacant and void of expression, and his breathing hurried; he could not speak, and there was a slight twist of the mouth. To take calomel, six grains; colocynth, ten grains; to be cupped on the temples to ten ounces. At night appeared to have derived no relief from the cupping; was sitting up in bed; countenance still vacant, but there was no paralysis; he took no notice when spoken to, and did not attempt to speak; pupils not dilated, but insensible; pulse feeble, skin perspiring. A turpentine injection was ordered. A catheter was introduced, and a considerable quantity of urine drawn off.

24th. No great change appeared to have taken place. He still continued sitting up in bed, not speaking, and taking no notice of anything passing around him; the pupils rather dilated; the pulse very feeble and rather quick: he was sick occasionally in the course of the day; there was no paralysis, but some degree of rigidity in the right arm; the catheter was still required. The head was shaved and a blister applied to the vortex. As there was great difficulty in getting him to swallow any medicine, some croton oil was given him on a little sugar. He continued getting gradually weaker, without any marked change of symptoms, until half-past three A. M. on the morning of the 25th, when he expired, sixty hours after the accident. According to the nurse's account, he "died very hard," struggling a good deal, and foaming at the mouth.

*Examination of the Body eleven hours after Death.*—There was no injury of the scalp or of the vault of the skull; no injury of the frontal bone beneath the wound. On removing the dura mater, lymph was found in large quantity spread over the whole upper surface of the brain beneath the arachnoid. On cutting into the brain, which was rather less firm than natural, pinkish in color, and very vascular, the lymph was found to dip in between the convolutions. A small portion of the substance of the brain at the posterior part of the right hemisphere, the point opposite to the wound on the left brow, was bruised and infiltrated with blood. The ventricles were filled with a considerable quantity of yellowish serum, containing numerous floating flocculi. The lining membrane of the ventricles was much injected. On removing the brain, a large quantity of sero-purulent fluid was found in the base of the skull. The medulla oblongata was very soft. There was a slight effusion of blood over the whole of the inferior surface of the left hemisphere. Puriform lymph thickly covered the whole of the inferior surface of the cerebellum, and was slightly spread over its superior surface. On removing the dura mater, there was found fracture of the cribriform plate of the ethmoid, and of the adjoining parts of the frontal bone. The fracture extended backwards through the anterior part of the sphenoid to its body. On reflecting the skin covering the root of the nose, the ossa nasi were found broken across at the upper part; and on pushing the nose upwards, the movement was communicated through the septum to the fractured cribriform plate. There was extensive emphysema of both lungs; the other viscera were healthy.

